# *Pseudomonas veronii* strain 7–41 degrading medium-chain *n*-alkanes and polycyclic aromatic hydrocarbons

**DOI:** 10.1038/s41598-022-25191-5

**Published:** 2022-11-28

**Authors:** S. A. Mullaeva, Ya. A. Delegan, R. A. Streletskii, O. I. Sazonova, K. V. Petrikov, A. A. Ivanova, I. A. Dyatlov, I. G. Shemyakin, A. G. Bogun, A. A. Vetrova

**Affiliations:** 1 Federal Research Center “Pushchino Scientific Center for Biological Research of the Russian Academy of Sciences”, G.K. Skryabin Institute of Biochemistry and Physiology of Microorganisms of the Russian Academy of Sciences, Pushchino, Moscow Region 142290 Russian Federation; 2 State Research Center for Applied Microbiology and Biotechnology, Obolensk, 142279 Russian Federation; 3 Faculty of Soil Science, Laboratory of Ecological Soil Science, Lomonosov Moscow State University, Moscow, 119991 Russian Federation

**Keywords:** Bioremediation, Pollution remediation

## Abstract

Pollution of the environment by crude oil and oil products (represented by various types of compounds, mainly aliphatic, mono- and polyaromatic hydrocarbons) poses a global problem. The strain *Pseudomonas veronii* 7–41 can grow on medium-chain *n*-alkanes (C_8_–C_12_) and polycyclic aromatic hydrocarbons such as naphthalene. We performed a genetic analysis and physiological/biochemical characterization of strain 7–41 cultivated in a mineral medium with decane, naphthalene or a mixture of the hydrocarbons. The genes responsible for the degradation of alkanes and PAHs are on the IncP-7 conjugative plasmid and are organized into the *alk* and *nah* operons typical of pseudomonads. A natural plasmid carrying functional operons for the degradation of two different classes of hydrocarbons was first described. In monosubstrate systems, 28.4% and 68.8% of decane and naphthalene, respectively, were biodegraded by the late stationary growth phase. In a bisubstrate system, these parameters were 25.4% and 20.8% by the end of the exponential growth phase. Then the biodegradation stopped, and the bacterial culture started dying due to the accumulation of salicylate (naphthalene-degradation metabolite), which is toxic in high concentrations. The activity of the salicylate oxidation enzymes was below the detection limit. These results indicate that the presence of decane and a high concentration of salicylate lead to impairment of hydrocarbon degradation by the strain.

## Introduction

Pollution of the environment by crude oil and oil products is a global problem^1,2^. Once in the soil, these components can be absorbed, concentrated and involved in trophic chains^3–5^. Cleaning the environment of petroleum hydrocarbons is not only an important environmental measure, but also contributes to improving the human environment^6,7^. The ability of microorganisms to degrade pollutants, including crude oil hydrocarbons (primarily *n*-alkanes and mono- and polyaromatic compounds), remains one of the most important topics of modern biotechnological research^8,9^. This ability makes it possible to use microorganisms in technologies for cleanup of the environment from crude-oil pollution^10,11^.

Bacteria of the genus *Pseudomonas* have a high degradation potential; they are capable of oxidizing a wide range of hydrocarbons including aliphatic^12,13^, mono-^14^, polyaromatic^15–17^, methylated and halogenated^18^ derivatives. Genes and biochemical pathways for the degradation of straight-chain alkanes and bi- and trinuclear aromatic hydrocarbons are well described^19^. The genetic organization of the *n*-alkane degradation system has been studied in detail as exemplified by the OCT plasmid of the *P. putida* strain GPo1^20^. Later, it was found that the chromosomal localization of these genes is more characteristic of pseudomonads^21,22^. The genes for the catabolism of mono- and polycyclic aromatic hydrocarbons (PAHs) in pseudomonads are usually located on large-size conjugative plasmids^23^.

All these results were obtained for microorganisms that degrade only one class of compounds, either alkanes or aromatic compounds. However, as early as in the 1970s, Chakrabarty et al. developed the *P. putida* strain NRRL B-5473 and *P. aeruginosa* strain NRRL B-5472 capable of degrading *n*-alkanes and PAHs^24^. Much later, natural pseudomonads possessing genes for the degradation of both classes of hydrocarbons were discovered^25^. Bacterial strains capable of degrading aliphatic and aromatic compounds were isolated for the first time by Whyte et al.^26^. *Pseudomonas* sp. strains BI7 and BI8 were able to grow on *n*-alkanes, toluene and naphthalene, as well as on a mixture of these substrates. An exhaustive review of bacteria capable of aerobic degradation of alkanes and aromatic hydrocarbons describes 20 strains of degrading pseudomonads^19^. Such strains have also been discovered in some recent works^27–30^. It is obvious that microorganisms capable of simultaneously oxidizing different classes of hydrocarbons occur in the environment. Probably, the degradative capabilities of microorganisms in relation to hydrocarbons of several different classes should provide them with a competitive advantage over strains degrading one class of compounds only^19^. Such microorganisms are of interest for fundamental research of biodegradative systems and have high potential for application in bioremediation technologies. The amount of research into this topic is, however, very limited. Most approaches to studies of pseudomonads capable of degrading alkanes and PAHs include merely the assessment of microbial growth ability on selected substrates, with some works analysing the genetic determinants responsible for hydrocarbon degradation^31^. It should also be kept in mind that the ability to grow on various classes of hydrocarbons is often simply not tested, which, obviously, reduces the significance of the described degraders. Our work proposes an approach that uses an extended assessment of the physiological parameters (construction of growth curves, substrate concentration) and biochemical characteristics (activity of key degradation enzymes, metabolite concentration) of the strain studied. The purpose of this work was to determine the genetic aspects of hydrocarbon biodegradation systems and to compare the physiological and biochemical characteristics of the *Pseudomonas veronii* strain 7–41^32,33^ in mono- and bisubstrate series of experiments.

## Results

### Nucleotide sequence and genetic organization of catabolic genes of the *P. veronii* 7–41 chromosome

**Figure 1:**
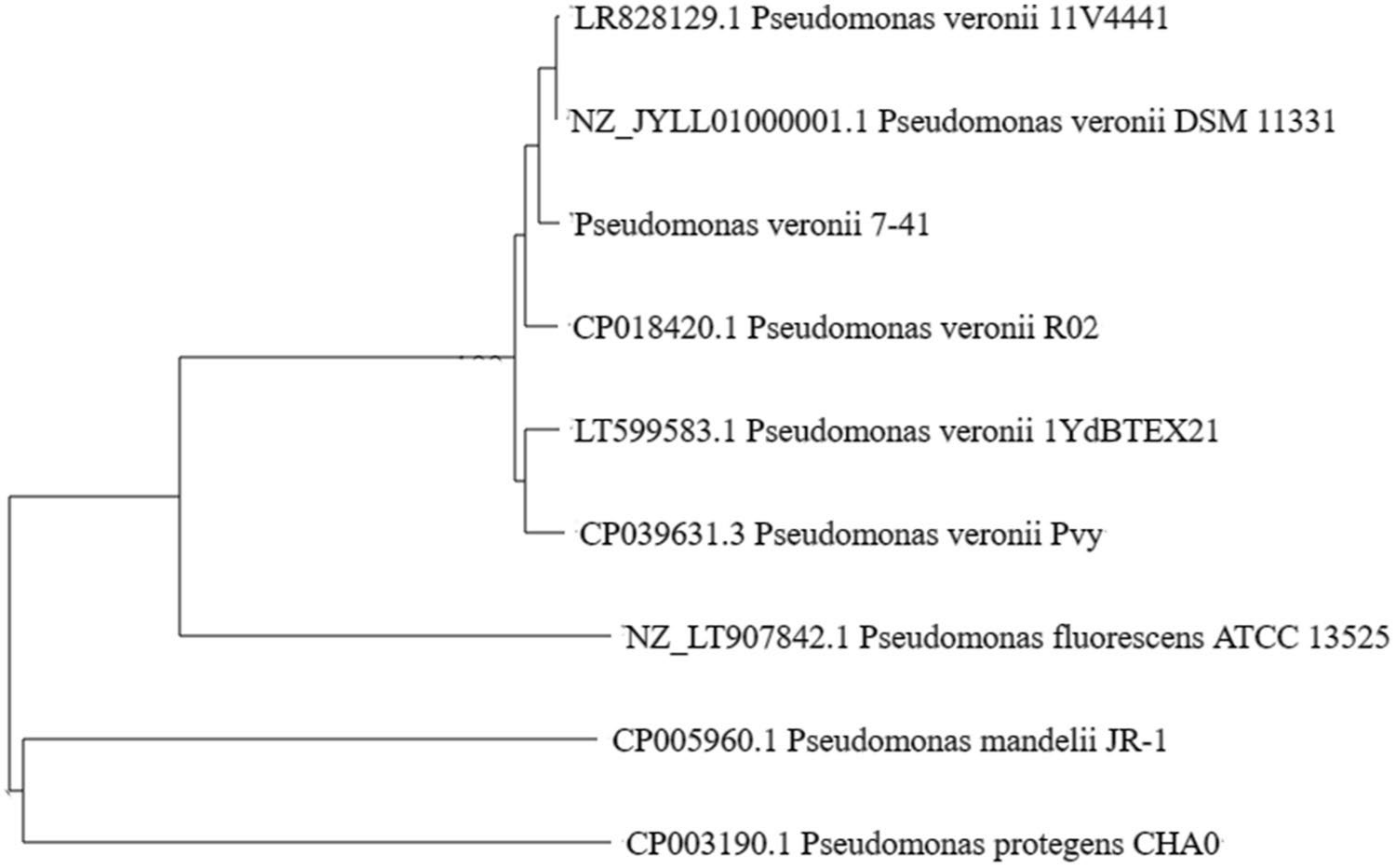
Neighbor‐joining phylogenetic tree of pseudomonads based on whole‐genome alignment.

Sequencing and complete assembly of the *P. veronii* 7–41 genome indicate that it has a circular chromosome replicon of 6,630,168 bp (GC content: 61.26%) (Fig. 1S).

**Figure 2:**
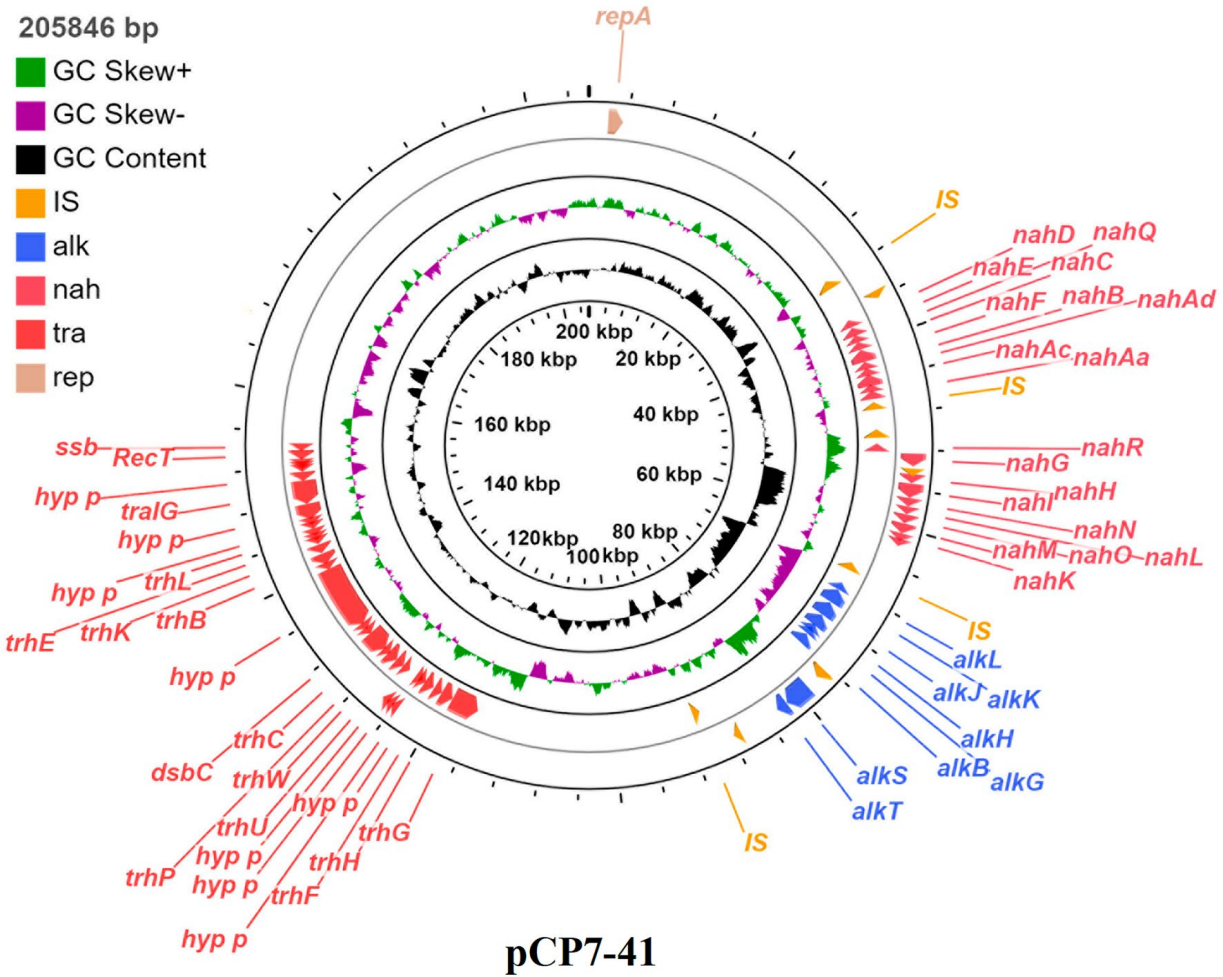
Circular map of the plasmid pCP7-41. From outside to the center: all CDS and RNA genes on the forward strand, all CDS and RNA genes on the reverse strand, GC content, and GC skew.

The chromosome contains 6014 coding sequences, 6 rRNA clusters, 1 tmRNA, and 68 tRNAs. A function was assigned to 3604 CDS, with 2341 CDS annotated as hypothetical proteins (Fig. 2S).

On the chromosome, we identified individual genes for alkane degradation, genes for decomposition of catechol via the *ortho*-cleavage pathway and genes for the degradation of other hydrocarbons (benzoate, aminobenzoate, toluene, styrene, and xylene).

To determine the taxonomic position of *P.veronii* 7–41 within the *Pseudomonas* genus, we compared the chromosome sequences of strain 7–41 with similar sequences of the closest related strains (Fig. 1) and calculated their DDH and ANI values (Table 1). We used the type strains of the species *P. veronii* DSM 11331, *P. fluorescens* ATCC 13525 and *P. protegens* CHA0.

**Table 1:** ANI and DDH values of the *P. veronii* 7–41 and related strains.

Strain	ANI, %	DDH, %
LR828129.1 *P. veronii* 11V4441	99.35	89.8
*CP018420.1 P. veronii R02*	98.95	83.4
CP039631.3 *P. veronii* Pvy	98.50	72.7
LT599583.1 *P. veronii* 1YdBTEX21	98.59	66.4
NZ LT907842.1 *P. fluorescens* ATCC 13525	87.58	49.7
CP003190.1 *P. protegens* CHA0	81.93	31.7
CP005960.1 *P. mandelii* JR-1	81.69	28.9
NZ JYLL01000001.1 *P. veronii* DSM 11331	99.21	15.8

**Figure 3:**
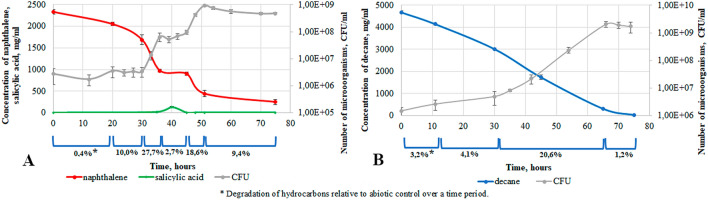
Growth parameters of the *P. veronii* 7–41 in naphthalene-MSEs (**A**) and decane-MSEs (**B**).

The phylogenetic tree (Fig. 1) confirms that the closest relative of strain 7–41 is *P. veronii* 11V4441 (LR828129.1; ANI, 99.35%; DDH, 89.8%). A Mauve-based comparison was performed between the chromosomes of *P. veronii* 7–41, *P. veronii* Pvy and *P. veronii* 1YdBTEX21 (Fig. 3S). The results obtained are characteristic of the genomes of *Pseudomonas* strains known for their instability and variability. Genome rearrangements in *Pseudomonas* promote adaptation, and a significant set of unique genes undoubtedly contributes to strain- and species-specific activity.

### Nucleotide sequence and genetic organization of catabolic genes of plasmid pCP7-41

DNA sequencing indicated that the size of pCP7-41 was 205,959 bp in length. The plasmid pCP7-41 has an overall G + C content of 54.37%.

This plasmid contains an origin of replication (*oriV*), a region involved in plasmid replication and stable inheritance, and regions involved in naphthalene and *n*-alkane catabolism (Fig. 2). This is the first report on a complete nucleotide sequence of a plasmid with naphthalene and *n*-alkane catabolic genes. In relation to the homology of other documented proteins and domains, putative functions were assigned to 30% of the coding sequence (CDS). Among them, 19 CDS were predicted to be involved in naphthalene catabolism; nine CDS in *n*-alkane catabolism; 16 CDS in conjugative DNA transfer; 15 CDS in transposition and integration; six CDS were related to proteins necessary for plasmid replication and partition and 51 were involved in other known or unknown functions.

#### Genes potentially involved in replication of plasmid

As mentioned above, six CDS most likely to be involved in replication and partitioning of the plasmid pCP7-41 were identified. The deduced amino acid sequence of the *repA* gene of pCP7-41 shows a high overall lengthwise identity (95–96%) with other IncP-7 plasmids. The deduced amino acid sequences of ORFs located upstream of the *repA* gene in pCP7-41 showed 71–95% identities to ParWABC proteins of pDK1 and 99–100% identities to corresponding proteins of pCAR1. These high identities suggest that the products of these ORFs function as partitioning proteins; these ORFs were designated as *parWABC*. Based on the nucleotide sequence of pCAR1 (AB088420), the possible origin of replication, *oriV*, is located immediately upstream of *repA*. The corresponding upstream region of *repA* of pCP7-41 (205,068–205,865 in CP089552) has a 99% and 84% identity on the nucleotide sequence level to the potential oriV of pCAR1 (70,202–70,987 in AB088420) and pDK1 (896–1776 in AB434906.1), respectively. Thus, the putative *oriV* region of pCP7-41 has almost the same structure as the pCAR1 *oriV* region and is located between the *repA* gene and the *parWABC* gene cluster. The product of ORF located downstream of *repA* has a 91% overall lengthwise identity with a potential DNA helicase of pCAR1. The deduced amino acid sequence of ORF, located further downstream of the potential DNA helicase, showed a 91% identity to the DNA replication terminus site binding protein of pCAR1. Thus, these CDS might also be involved in the replication of pCP7-41.

Plasmids are classified into incompatibility (Inc) groups based on their replication and partition systems: two plasmids belonging to the same Inc group are unable to coexist in one bacterial cell^34^. The pCP7-41 was assigned to IncP-7 only on the basis of PCR results^32^. The replication systems (*oriV* and *repA* genes) and partition systems (*parWABC*) of pCP7-41 show a significant identity with those of other known IncP-7 plasmids, especially with pCAR1. Members of the same Inc group have a common plasmid backbone determining their basic features. Thus, the plasmid pCP7-41 does belong to the incompatibility group P-7.

#### Organization of n-alkane and naphthalene degradation genes

The plasmid pCP7-41 contains all the genes required for the oxidation of alkanes to acyl-CoA derivatives. These genes are organized into 2 operons, *alkST* and *alkBFGHJKL*, and are located in a region of about 23 kb. The organization and mutual arrangement of clusters of alkane degradation genes of the *P. veronii* strain 7–41 are identical to those of the *P. putida* strain P1^20^; however, the *alk* genes in the latter have a chromosomal localization. The identity of the nucleotide and deduced amino acid sequences of most *n*-alkane degradation genes of the plasmid pCP7-41 with the corresponding sequences of the *P. putida* strain P1 and *P. veronii* VI4T1 is 100% (Table 1S). Exceptions are the *alkB* and *alkK* genes, for which the level of DNA sequence identity is 99%. Analysis of the deduced amino acid sequence of the *alkK* gene of the plasmid pCP7-41 made it possible to establish that the substitution of cytosine for thymine at position 715 of the nucleotide sequence of this gene results in the substitution of histidine for tyrosine in the amino acid sequence of the protein. As in the *P. putida* strain P1, clusters of *alk* genes of the plasmid pCP7-41 are flanked by identical copies of the insertion sequence IS*Ppu4*, constituting a class 1 transposon (Tn*Ppu-alk1*).

Most of the currently described naphthalene and salicylate biodegradation genes in pseudomonads have a conservative organization and a high degree of homology (about 90%) with the *nah* genes of the NAH7 plasmid of *P. putida* G7^35–38^. The structure and DNA sequence of the *nah* operons on pCP7-41 were compared with other complete operons involved in the degradation of naphthalene.

The pCP7-41 naphthalene degradation genes are located upstream of *alk* genes in two gene clusters of 36,911 bp and 61,526 bp (bp 36,911–46,198 and 52,326–61,526, respectively). This represents 12% of the total pCP7-41 DNA sequence.

The structure of the catabolic operons of pCP7-41 is similar to that of the plasmids pND6-1 (AY208917.2), pDTG1 (AF491307.2) and NAH7 (NC_007926.1). The upper pathway operon (*nah* operon) contains nine genes (*nahAaAbAcAdBFCED*) encoding enzymes for the conversion of naphthalene to salicylate. The other 10 genes (*nahGTHINLOMKJ*) form the lower pathway operon (*sal* operon), which encodes the enzymes for the conversion of salicylate to tricarboxylic acid cycle intermediates through the enzymes of the *meta*-cleavage pathway.

The identity of the nucleotide sequences of the *nah* and *sal* genes of the operons of the plasmid pCP7-41 with the corresponding sequences of the naphthalene catabolic plasmid pND6-1 (AY208917.2), an unnamed plasmid of *P. veronii* strain Pvy (CP039632.3), the plasmid pDTG1 (AF491307.2) and the plasmid NAH7 (NC_007926.1) is 77–94%. The deduced amino acid sequences of these ORFs showed 77–95% identities (Table 1S).

The deduced amino acid sequence of the ORF located upstream of the *nahG* gene showed a high degree of homology (95%) with the corresponding sequence of the unnamed plasmid of *P. veronii* strain Pvy (CP039632.3), which encodes a LysR-type transcriptional regulator for the expression of naphthalene and salicylate catabolic operons, and an 83–85% identity with the *nahR* gene of pND6-1, pDTG1 and NAH7.

The organization of clusters of naphthalene degradation genes of the plasmid pCP7-41 is identical to that of the plasmid NAH7; however, the sequence containing the *nah* operon is inverted with respect to the *sal* operon and is transcribed in the opposite direction. The cluster of genes of the plasmid pCP7-41 constituting the naphthalene degradation upper pathway is flanked by IS elements of the IS*110* family transposase.

#### Conjugative transfer functions

The conjugative systems of many plasmids of Gram-negative bacteria consist of three components: the mating pair formation apparatus, the relaxosome and the coupling protein^39^. The transfer functions of pCP7-41 are encoded in a 49.5 kb segment of the plasmid backbone that includes all of the abovementioned components. The genes in this region of DNA are arranged identically to those of the IncP-7 plasmids pCAR1 and pDK1, and the amino acid residues of the gene products range from an 81% to 100% identity. In addition to the 15 conjugation genes found in this region, it also contains a putative *oriT* site identifiable by its similarity to other *oriT* sequences including that of pCAR1 and pDK1. Other experimental approaches are required to elucidate the location of the *oriT* site of pCP7-41.

The region of pCP7-41 from *trhN* to putative *oriT* was highly conserved in pCAR1 and pDK1 except for some insertion/deletion polymorphism. Namely, pDK1 contains the IS200/IS605 transposase gene and the *ofn40* region, which are absent in pCP7-41 and pCAR1. In addition, pCAR1 has an interruption in the 134,607–144,966 region (AB088420.3) by the IS*Pre3* insertion, which is not observed either in pCP7-41 or in pDK1. Another distinguishing feature is the absence, between the *traI* and *recT* genes in pCP7-41, of the sequences of the corresponding *ofn38* and *ofn39* of the pDK1 plasmid or IS*Pre4* in pCAR1. Despite these polymorphisms, pCP7-41 retained its self-transmissibility.

Experiments on the conjugative transfer of the pCP7-41 plasmid into plasmid-free recipient strain KT2440 showed this plasmid to contain all the necessary genes for the transformation of naphthalene and *n*-alkanes into Krebs cycle intermediates. The conjugative transfer frequency was 10^−6^–10^−7^ per donor cell.

**Figure 4:**
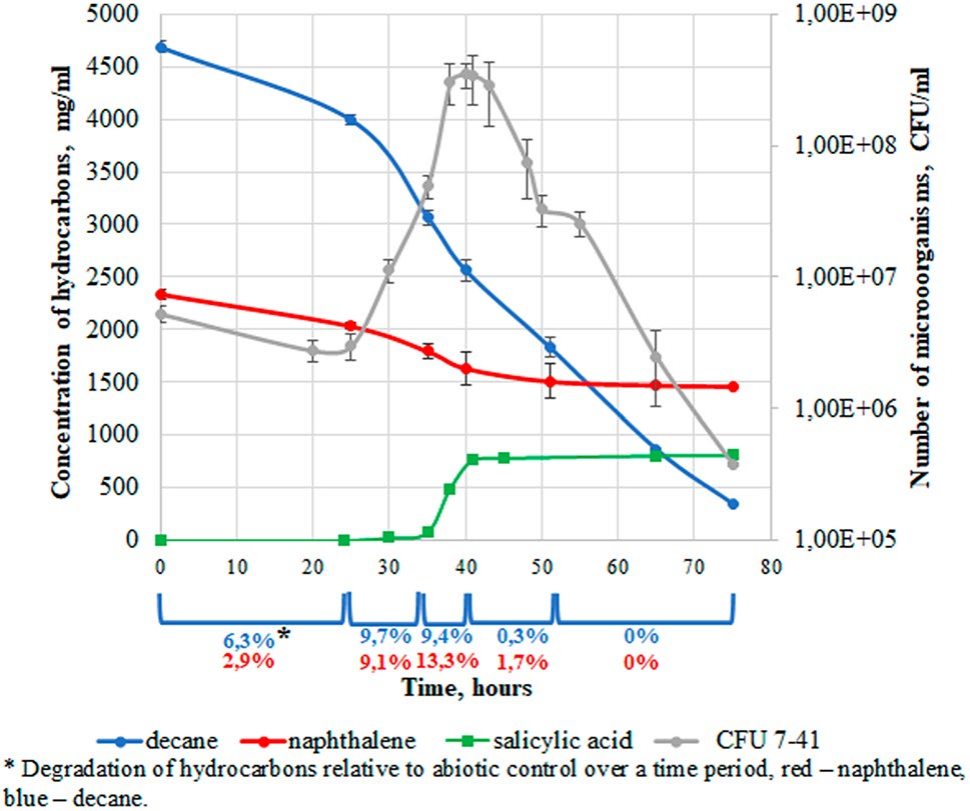
Growth parameters of the *P. veronii* 7–41 in BSEs.

A Mauve-based comparison was performed between the plasmid pCP7-41 of *P. veronii* 7–41, the pDK1 of *P. putida* HS1 and the pCAR1 of *P. resinovorans* strain CA10 (Fig. 4S). All plasmids studied contain genes for the catabolism of aromatic hydrocarbons. The ANI value of two plasmids (pCP7-41/pCAR1 and pCP7-41/pDK1) showed a high degree of identity of their sequences (ANIs, 97.39% and 92.8%, respectively).

### Growth of *P. veronii* 7–41 in mineral medium with* n-*decane and naphthalene at 26 °C

Strain 7–41 was cultivated in a liquid mineral medium at 26 °C in monosubstrate experiments with either *n*-decane or naphthalene (decane-MSEs and naphthalene-MSEs; Fig. 3) and in bisubstrate experiments with *n*-decane and naphthalene (BSEs; Fig. 4).

**Figure 5:**
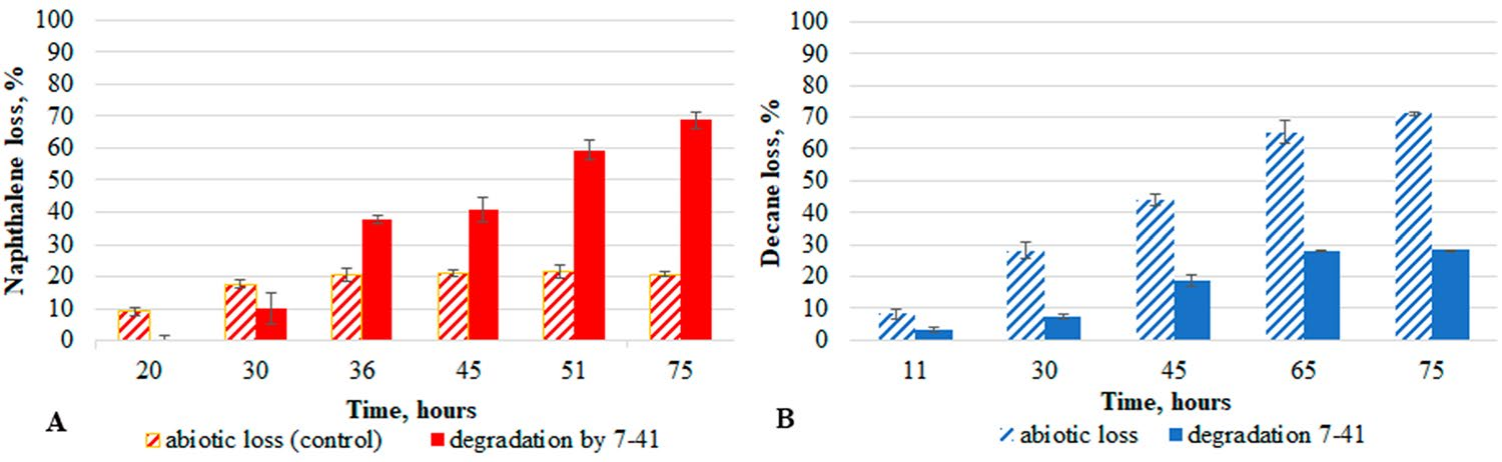
Abiotic loss (shaded columns) and degradation of hydrocarbons by *P. veronii* 7–41 in the naphthalene-MSEs (**A**) and decane-MSEs (**B**) at a temperature of 26 °C.

**Figure 6:**
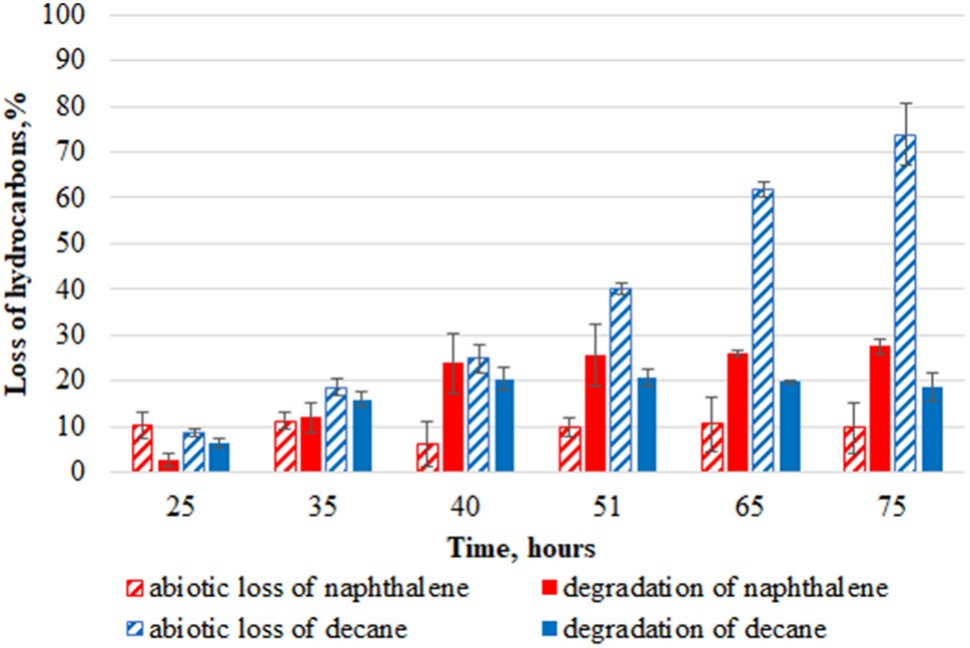
Abiotic loss (shaded columns) and degradation of hydrocarbons by *P. veronii* 7–41 in BSEs (naphthalene-red and decane-blue) at a temperature of 26 °C.

The growth curve in naphthalene-MSEs had a two-step form, which is indicative of the formation of a metabolite (salicylate, about 150 mg/l), which temporarily inhibits microbial growth (Fig. 3A). At the maximum concentration of salicylate, we detected catechol (about 0.30 mg/l) in the medium; it was not detectable during the subsequent hours of growth. The histograms in Figs. 5 and 6 show the cumulative increase of the abiotic loss in control systems without microorganisms and the sum total of the abiotic loss and the degradation in the analysed systems with strain 7–41. During the first 30 h of bacterial growth, the abiotic loss was greater as compared with naphthalene degradation by strain 7–41. In the next hours of culture growth, the opposite was observed, the abiotic loss reached 20% and did not change (Fig. 5A). The total degradation of naphthalene by strain *P. veronii* 7–41 relative to the abiotic control was 68.8% after 75 h. At the same time, the largest fraction of the substrate was degraded during the culture growth periods of 30–36 h (27.7%) and 45–51 h (21.7%), which correlates with the data of the specific microbial growth rate (µ_30–36_ = 0.49 h^−1^, µ_45–51_ = 0.39 h^−1^). The substrate uptake rate during these periods was 89.3 mg/ (l × h) and 74.5 mg/ (l × h; Table 2).

**Table 2:** Physiological parameters of strain growth in MSEs and BSEs.

Experimental series	Time of period, hours	Substrate consumption rate in a certain period of time (υ), [mg/(l × hour)]	Microbial culture specific growth rate (µ)	Total degradation of hydrocarbons, %
Naphthalene-MSEs	30–36	89.3 ± 4.2	0.49 ± 0.02	68.8 ± 3.4
	45–51	74.5 ± 2.7	0.39 ± 0.02	
Decane-MSEs	30–66	26.8 ± 1.3	0.17 ± 0.01	28.4 ± 1.4
BSEs	25–40	70.3 ± 3.2 (44.1 ± 2.2 decane) (26.2 ± 1.3 naphthalene)	0.32 ± 0.01	18.7 ± 0.9 decane 27.5 ± 1.4 naphthalene

The growth curve in decane-MSEs had a classical S-shape. A significant abiotic loss throughout the experiment was noted, which is consistent with the data of decane’s volatility. The degradation of decane by strain 7–41 during the exponential growth phase of the culture was about 20.6%, which correlates with the gradual growth curve and a lower specific growth rate (µ_30–66_ = 0.17 h^−1^). The total degradation of decane by strain 7–41 by 75 h was about 30%. The substrate uptake rate between 30 and 66 h of growth was 26.8 mg/(l × h; Table 2).

Comparison of the degradation of naphthalene and decane in MSEs showed a higher rate of PAH degradation. However, the naphthalene-MSEs experiment revealed a temporary accumulation of a secondary metabolite, salicylate, which had an inhibitory effect on culture growth and further consumption of naphthalene (Fig. 3A).

In BSEs, there was a decrease in the number of microorganisms after 43 h, possibly also related to the accumulation of salicylate in the medium at a concentration of about 770 mg/l. At 35 h of growth, an insignificant amount of catechol (0.35 mg/l) was detected in the medium; it did not change until the end of the experiment. The specific microbial growth rate in BSEs was µ_25–40_ = 0.36 h^−1^. The level of abiotic loss of naphthalene in the control system (Fig. 6) practically did not change throughout the experiment (about 10%). The degree of decane degradation in the first 25 h was two times higher compared to that for naphthalene. The total rate of consumed substrates in the period from 25 to 40 h was 70.3 mg/ (l × h); for decane, it was 44.1 mg/ (l × h); for naphthalene, 26.2 mg/ (l × h; Table 2). In BSEs the total degree of degradation for decane decreased by 1.5 times (2.5 times for naphthalene) compared with MSEs.

The activities of the key enzymes of hydrocarbon degradation (Table 3) correlate with the growth and substrate consumption curves. The activities of the enzymes studied were inducible, especially in relation to salicylate hydroxylase and catechol 2.3-dioxygenase, which were absent when the strains were grown on succinate. The activities of alkane hydroxylase and naphthalene 1.2-dioxygenase did not change both in BSEs and in corresponding MSEs. The activities of salicylate hydroxylase and catechol 2.3-dioxygenase were not detected during salicylate accumulation in naphthalene-MSEs but were significant in the subsequent hours of growth. The maximum value of salicylate hydroxylase activity in BSEs was 24 μmol × mg^−1^ proteins, which was 40 times less than the maximum value of this parameter in naphthalene-MSEs. The specific activity of salicylate hydroxylase was not detected after 40 h of culture growth in BSEs. In decane-MSEs, the specific activity of salicylate hydroxylase was not detected. The activities of catechol 1.2-dioxygenase and catechol 2.3-dioxygenase were undetectable in both decane-MSEs and BSEs.

**Table 3:** Activity of the key enzymes of naphthalene and decane degradation.

Substrate	Enzyme activity, μmol × mg^−1^ protein				
	Naphthalene 1.2-dioxygenase	Salicylate hydroxylase	Catechol 1.2-dioxygenase	Catechol 2.3-dioxygenase	Alkane hydroxylase
Naphthalene	41 ± 3 (36; 51)*	˂ 1 (36) 1002 ± 195 (51)	28 ± 8 (36; 51)	˂1 (36) 1053 ± 239 (51)	12 ± 5 (36; 51)
Succinate**	15 ± 3	˂ 1	8 ± 2	˂ 1	13 ± 5
Decane	23 ± 8 (50; 66)	˂ 1 (50; 66)	˂ 1 (50; 66)	˂ 1 (50; 66)	28 ± 2 (50; 66)
Decane and naphthalene	45 ± 5 (35; 40)	24 ± 3 (35) ˂ 1 (40)	˂ 1 (35; 40)	˂ 1 (35; 40)	24 ± 3 (35; 40)

*Time, h.

**Enzyme activity was measured in the biomass of cells sampled at the end of the exponential growth phase.

### Growth of *P. veronii* 7–41 in mineral medium with* n-*decane and salicylate at 26 °C

Salicylate concentrations in naphthalene-MSE and BSEs reached 0.2 g/l and 0.8 g/l, respectively. Strain 7–41 was cultivated in a liquid mineral medium at 26 °C in monosubstrate experiments with salicylate (salicylate-MSEs) and in bisubstrate experiments with *n*-decane and salicylate (SD-BSEs; Table 4). These experiments were carried out to study the effect of different concentrations of salicylate on the growth of the strain. Increasing the salicylate concentration in salicylate-MSEs and SD-BSEs led to lag phase lengthening. The presence of decane in SD-BSEs also contributed to an almost two-fold increase in the lag phase. The culture death was detected at 0.8 SD-BSE. Apparently, the concentration of salicylate above 0.60 g/l in SD-BSE had an inhibitory influence on the culture growth.

**Table 4:** Salicylate concentration during strain cultivation in salicylate-MSEs and SD-BSEs for 5 days.

Time, h	Salicylate concentration, g/l					
	0.2 Salicylate-MSEs	0.2 SD-BSEs	0.6 Salicylate-MSEs	0.6 SD-BSEs	0.8 Salicylate-MSEs	0.8 SD-BSEs
0	0.20	0.20	0.60	0.60	0.80	0.80
26	0.00 (10^7^ CFU/ml)*	0.20	0.60	0.60	0.80	0.80
47	0.00	0.06	0.40	0.60	0.80	0.80
69	0.00	0.00 (10^9^ CFU/ml)	0.05	0.60	0.64	0.80
90	0.00	0.00	0.00 (10^8^ CFU/ml)	0.34	0.02	0.80
120	0.00	0.00	0.00	0.00 (10^8^ CFU/ml)	0.00 (10^8^ CFU/ml)	0.80 (ND**)

*Concentration of bacterial cells by the time of salicylate complete consumption.

**ND not detected.

## Discussion

The present study clarified that strain 7–41 was definitively attributed to the species *Pseudomonas veronii*. Analysis of the NCBI data showed it to be the fourth strain of the species *Pseudomonas veronii* with a complete genome assembly.

The ability of natural pseudomonads to simultaneously degrade different types of substrates has been known for more than 30 years. Earlier, Whyte et al.^26^ isolated a strain of the *Pseudomonas* genus capable of simultaneous degradation of aliphatic and aromatic hydrocarbons. The genes encoding the degradation enzymes of these compounds were shown to be located on two different plasmids. The presence of genes for the degradation of PAHs on plasmids is a widespread phenomenon in pseudomonads, in contrast to genes for the degradation of aliphatic hydrocarbons, which often have a chromosomal localization. Until now, the genes for alkane degradation localized on the plasmid have been identified only in the *Pseudomonas* sp. strain BI7^26^ and *P. putida* strain GPo1 with the conjugative plasmid OCT^20^. The present study shows that in strain 7–41 the *nah* and *alk* genes were localized on the same plasmid. The conjugative transfer of the plasmid pCP7-41 from the *P. veronii* strain 7–41 to the *P. putida* strain KT2440 confirmed the functionality of the plasmid biodegradation genes. It should be noted that plasmids, which simultaneously carry genes for assimilation of alkanes and PAHs, have not been previously found in microorganisms of the genus *Pseudomonas*.

A detailed study of the physiological and biochemical parameters of the growth of strains on a mixture of substrates and a comparison of these characteristics with growth on individual substrates has not been practically studied. In our opinion, research into the degradation processes of different classes of hydrocarbons in bisubstrate systems by degraders of aromatic and aliphatic compounds is important because it can expand knowledge on oil hydrocarbons’ degradation to improve biotechnology for elimination of technogenic pollution in the environment.

For this reason, we decided to compare the physiological and biochemical components of the degradation of different classes of hydrocarbons by *P. veronii* strain 7–41 in bisubstrate and monosubstrate systems.

This work shows that the activities of the key enzymes of the naphthalene degradation lower pathway, salicylate hydroxylase and catechol 2.3-dioxygenase, were not detected during the first 35 h of growth in naphthalene-MSE. This led to accumulation of salicylate in the medium. A similar effect was observed by Filonov et al.^40^. At the same time, the activity of naphthalene 1.2-dioxygenase, a key enzyme of the upper pathway of naphthalene degradation to salicylate, was comparable to the activity level of this enzyme when the strain was grown on succinate. This indicates that NahA is probably constitutively expressed in *P. veronii* 7–41 at the low level. From 35 to 45 h of cultivation, the growth of the strain was slowed. It is known that the second operon of PAH degradation is responsible for the oxidation of salicylate, and salicylate is an inducer of both *nah* and *sal* operons^41^. Probably, to activate the transcription of naphthalene degradation operons, strain 7–41 should accumulate a certain concentration of salicylate in the medium that, in turn, has an inhibitory effect on the growth of the bacterial strain. Similar effects of salicylate on microorganisms are well known^42^. Further secondary growth of the culture is probably related to the activation of the gene expression of the lower pathway, the subsequent consumption of salicylate, which was expressed in a decrease of its concentration in the medium and further induction of *nah* operon. Both catechol 2.3-dioxygenase and salicylate hydroxylase activity appeared during this period, and naphthalene 1.2-dioxygenase activity increased. A similar effect was not detected when the culture was grown on a medium with succinate. This indicates that catechol 2.3-dioxygenase and salicylate hydroxylase are inducible enzymes.

In decane-MSEs, the abiotic loss of substrate significantly exceeded the microbial degradation of *n-*alkane throughout the experiment, and the insolubility of decane in the medium reduced the amount of available substrate. As a result, the duration of the exponential growth phase on decane was about 36 h, whereas in naphthalene-MSEs it was shorter (6 h). Comparison of monosubstrate systems showed the strain to be a more effective PAH degrader because the uptake rate and total level of naphthalene degradation by the *P. veronii* strain 7–41 were three and 2.5 times higher compared to those for decane.

The abiotic loss of PAHs in BSEs was two times lower compared to naphthalene-MSEs due to the presence of a hydrophobic decane film on the surface of the liquid mineral medium, which decreases sublimation of naphthalene. Probably, in the BSEs, the sublimation of naphthalene was significantly reduced due to the dissolution of sublimated naphthalene in hydrophobic decane localized on the surface of the medium as the film, thereby delaying naphthalene in the system.

Sotsky et al.^25^ suggested the presence of a certain regulation providing a selective expression of genes encoding enzymes for the degradation of different classes of hydrocarbons. Whyte et al.^26^ studied the degradation of a mixture of naphthalene, octane, and toluene by the *Pseudomonas* sp. strains BI7 and BI8. They found that both strains, when cultivated on a mixture of hydrocarbons, first consumed naphthalene and then octane and toluene, which was confirmed by both the rate and the degree of biodegradation of the three compounds. In the present study, another phenomenon was observed in BSEs: the *P. veronii* strain 7–41 consumed both substrates. In BSEs it was found that the rate of alkane degradation was two times higher than that of naphthalene in the first 25 h. This is consistent with the data of Huesemann^43^ that *n-*alkanes are the highest priority substrates for microorganisms compared to other oil hydrocarbons, including aromatic compounds. After 35 h of cultivation in BSEs, the degrees of naphthalene and decane degradation were almost levelled up, and the degree of naphthalene degradation exceeded the degree of decane degradation in the period from 35 to 50 h. Apparently, this was related with a high level of *n*-alkane abiotic loss during the entire experiment. It should be noted that neither naphthalene nor decane by themselves had a negative influence either on the culture growth or on the hydrocarbon’s degradation in BSEs. This was confirmed by the first 30 h of the growth curve of strain 7–41 in BSEs (Fig. 4), as well as by the presence of naphthalene 1.2-dioxygenase and alkane hydroxylase activities in the decane-MSEs and naphthalene-MSEs, respectively.

In BSEs (from 38 to 43 h), as well as in naphthalene-MSEs (from 36 to 45 h), periods of no increase in cell biomass were revealed. This period of time is characterized by the salicylate accumulation in these systems. However, in BSEs, the concentration of accumulated salicylate (about 6.5 mM) was found to be four times higher compared to naphthalene-MSEs. Moreover, in BSEs, the absence of activity of catechol 1.2-dioxygenase and catechol 2.3-dioxygenase enzymes and low activity of salicylate hydroxylase compared to MSE were revealed. As a result, the accumulation of salicylate in BSEs led to growth inhibition and subsequent death of the culture. It can be noted that the negative effect of salicylate concentration was revealed in the work of Jõesaar et al.^44^ as the growth of the *Pseudomonas pseudoalcaligenes* strain C70 was inhibited at a salicylate concentration of 3.4 mM in a mineral medium^44^.

It was suggested that one of the critical factors may not be the presence of salicylate in the medium itself, but its concentration. To confirm the hypothesis put forward, an experiment was conducted with different concentrations of salicylate in salicylate-MSEs and SD-BSEs (Table 4). It was found that with an increase in salicylate concentration, the growth lag phase of the *P. veronii* 7–41 strain in salicylate-MCEs was lengthened, and the additional presence of the decane contributed to an increase in the period of adaptation by another two times in SD-BSEs. That is, a synergistic effect of the influence of the salicylate concentration and the presence of decane in SD-BSEs on the growth of the strain and the process of hydrocarbons degradation was revealed.

The accumulation of salicylate and the absence of its degradation in the medium in BSE indicate a disturbance of the process of degradation of salicylate in the bisubstrate system. We suggest several options for possible causes of the observed phenomenon. In the presence of decane, the regulation of the expression of *sal* operon genes encoding the corresponding enzymes changes at the level of transcription or translation. It is also possible that decane or its metabolic products somehow affect the active centres of these enzymes. It is possible that there are mechanisms of negative and positive regulation, as shown previously in the study of lactose metabolism regulation in *E. coli*. In addition, salicylate and decane are known to influence changes in the properties of the cell membrane. In particular, the presence of these compounds in the medium can lead to changes in membrane fluidity, repression of transcription of porin-coding genes and inhibition of efflux pumps^42,45^. In the next work, it is planned to use transcriptomic and proteomic analyses to identify the true cause of the mechanism of hydrocarbon degradation by the *P. veronii* strain 7–41 in BSEs.

Thus, the approach described in the “Introduction” section was used to compare the culture characteristics of *P. veronii* strain 7–41 in MSEs, each with one of the two chosen hydrocarbons and in BSEs. However, the simultaneous presence of genes for the degradation of alkanes and PAHs in the microorganism did not contribute to an increase in the efficiency of degradation of different classes of hydrocarbons by the strain under study. In BSEs the metabolism of salicylate was hindered, which led to its accumulation and the rapid death of bacteria. The cumulative effect of the aliphatic compound and a key metabolite of naphthalene (salicylate) on the process of hydrocarbons degradation in BSEs was revealed. This shows a fundamental difference in the regulation of the degradation of compounds by a degrader in MSEs compared with BSEs containing hydrocarbons of different classes.

The use of a strain capable of simultaneous degradation of different classes of hydrocarbons as an alternative to microbial consortiums for cleaning the environment from crude oil may be ineffective. However, it is possible to use *P. veronii* 7–41 as part of a consortium for cleaning the environment from hydrocarbons, provided that one of the members of the proposed consortium will be able to degrade the accumulated secondary metabolite (salicylate). The obtained results indicate the need to study the influence of one class of hydrocarbons on the mechanism of degradation of other microorganisms capable of degrading different classes of hydrocarbons and by degraders of individual compounds.

## Conclusion

The *Pseudomonas veronii* strain 7–41 has a conjugative catabolic plasmid IncP-7 carrying genes for the degradation of medium-chain *n*-alkanes and naphthalene. It is the first described natural pseudomonad plasmid carrying functional operons for the degradation of two different classes of hydrocarbons. The combined negative effect of an aliphatic compound and a secondary metabolite on the hydrocarbons degradation in the bisubstrate system was first shown on the example of a strain of aromatic and aliphatic hydrocarbons degradation. A detailed study of the interaction between the metabolic systems for alkanes and PAHs would require further work involving transcriptomics and proteomics, which will make it possible to carry out a comprehensive assessment of changes in the expression of all genes and proteins involved in the utilization of hydrocarbons of different classes. Acquired knowledge will contribute to the improvement of biotechnological approaches to the elimination of man-made environmental pollution.

## Materials and methods

### Microorganism

The strain *Pseudomonas* sp*.* 7–41 was isolated from the oil-contaminated saline soil of the Middle Ob region^32,33^. The strain is capable of degrading aliphatic (C_7_–C_11_) and aromatic hydrocarbons (naphthalene, salicylate (0.02 g/l)) in a wide temperature range (4–30 °C) with an optimum growth at 26 °C. This strain catabolizes hydrocarbons in a wide pH range (4.5–8) with the optimal value of pH 7. Also, we used the strain *Pseudomonas putida* KT2440 (AE015451.2) containing the *gfp* (green fluorescence protein) gene.

### Chemicals

High purity grade (> 98%) naphthalene and decane were from Sigma-Aldrich (USA). Analytical grade dichloromethane was purchased from Sigma-Aldrich (USA). All the other reagents were of analytical grade.

### Growth media and conditions

The strain *Pseudomonas* sp. 7–41 used in this study was grown at 26 °C on a modified Evans mineral medium supplemented either with succinate (2 g/l) or naphthalene (2 g/l) or decane (6 ml/l) or salicylate (0.2 g/l, 0.6 g/l, 0.8 g/l) or both naphthalene (2 g/l) and decane (6 ml/l) or both salicylate (0.2 g/l, 0.6 g/l, 0.8 g/l) and decane (6 ml/l) as the sole source of carbon and energy. The modified Evans mineral medium contained (per liter) 50 mM K_2_HPO_4_, 5 mM NH_4_Cl, 0.1 mM Na_2_SO_4_, 0.0625 mM MgCl_2_, 0.018 mM FeCl_3_, 0.01 mM MnCl_2_, 0.005 mM ZnO, 0.002 mM CoCl_2_, 0.001 mM CaCl_2_, 0.97 μM CuCl_2_, 0.97 μM H_3_BO_3_, and 0.005 μM (NH_4_)_6_Mo_7_O_24_^46^. The pH of the medium was adjusted to 7.5 using concentrated HCl.

To assess the dynamics of bacterial growth and to obtain single colonies of pure culture, we used a Lysogeny Broth (LB) agar medium^47^ consisting of 10 g/l tryptone, 5 g/l yeast extract, 5 g/l NaCl (in distilled water) and 20 g/l agar (Difco, USA).

The seed culture was prepared by inoculating cells grown on plates into tubes containing 10 ml of the medium (pH 7.5) supplemented with 2% (w/v) succinate, which was followed by incubation at 26 °C for 24 h with agitation in an orbital shaker at 180 rpm. Cells were harvested by centrifugation at 10,000 rpm for 10 min at 4 °C and resuspended in 10 ml of a mineral medium at a final concentration of 1 × 10^8^ CFU/ml. The inoculum was introduced into flasks so that the initial concentration of cells in the medium was about 5 × 10^6^ CFU/ml.

The assessment of the dynamics of bacterial growth, substrate consumption, and the measurement of enzymatic activity was carried out in 750-ml Erlenmeyer flasks.

The flasks contained 100 ml of a mineral medium supplemented with naphthalene (2 g/l) (naphthalene-MSEs) or decane (6 ml/l) (decane-MSEs) or else both naphthalene (2 g/l) and decane (6 ml/l) (BSEs) as the sole source of carbon and energy. The flasks were cultured at 26 °C for about 4 days in an orbital shaker at 180 rpm.

The flasks contained 100 ml of a mineral medium supplemented with salicylate (0.2 g/l, 0.6 g/l, 0.8 g/l) (0.2 salicylate-MSEs, 0.6 salicylate-MSEs, 0.8 salicylate-MSEs) or else both salicylate (0.2 g/l, 0.6 g/l, 0.8 g/l) and decane (6 ml/l) (0.2 SD-BSEs, 0.6 SD-BSEs, 0.8 SD-BSEs) as the sole source of carbon and energy. The flasks were cultured at 26 °C for about 5 days in an orbital shaker at 180 rpm.

All results are derived from three independent biological replicates.

### Genome sequencing, assembly and annotation

Genomic DNA was extracted from the biomass of a fresh culture of *Pseudomonas* sp. 7–41 grown on LB agar using the Wizard Genomic DNA Purification Kit (Promega, USA). Sequencing was performed using a MinION sequencer with the FLO-MIN106 flow cell (Oxford Nanopore Technologies [ONT]). A library was prepared with a ligation sequencing kit (catalog number SQK-LSK109). The Guppy version 3.2.4 software was used for base calling, which yielded a total of 293 Mb distributed in 61,481 reads with a score Q > 10.

The same DNA sample was sequenced with an Illumina NovaSeq 6000 platform using an S2 reagent kit (catalog number 20012861; 2 × 100 bp). A paired-end library was prepared with the KAPA HyperPlus Kit (KAPAbiosystems). The Illumina and Nanopore reads were used for hybrid assembly with the SPAdes version 3.15.2^48^. The Nanopore reads were assembled into contigs using the Flye assembler version 2.6^49^. The SPAdes contigs were then combined into replicons using Flye data as reference. The Illumina reads were used to correct Nanopore errors using the Bowtie2 version 2.3.5.1^50^ and Pilon version 1.23^51^ software. Circularization of the ends of replicons (chromosomes and plasmids) was confirmed by overlapping ends.

The data were submitted to the GenBank database under the following accession numbers: BioProject, PRJNA783734; BioSample, SAMN23443333; GenBank, NZ_ CP089551- NZ_ CP089551.

The assembled genome was annotated using Prokka^52^. Functional pathways were interpreted using the Kyoto Encyclopedia of Genes and Genomes (KEGG)^53^ by using the blastp module. The functions of some proteins were checked manually using the Basic Local Alignment Search Tool (BLAST)^54^. The following processes and genome elements were specifically examined: aromatic and aliphatic compounds’ degradation.

The circular maps were made using CGView^55^. A genome alignment performed with Mauve^56^ between our strain and the type strain was carried out, showing the similarity and conserved synteny of genes.

The phylogenetic tree was constructed by the neighbor‐joining method using the REALPHY service^57^. Genome sequences of *Pseudomonas* strains required for constructing the phylogenetic tree were taken from NCBI (https://www.ncbi.nlm.nih.gov/genome/browse/#!/prokaryotes/17302/). For taxonomic purposes, the OrthoANI was calculated between the genomic DNA of strain 7–41 and related strains using the OrthoANI algorithm^58^. DDH was calculated using the Genome-to-Genome Distance Calculator 2.1 service^59^.

### Conjugative transfer of the bacterial plasmid and assessment of phenotypic traits’ stability

The conjugative transfer of the plasmid pCP7-41 from the *Pseudomonas veronii* strain 7–41 was carried out using a modified method of Dunn and Gonzalez^36^. *Pseudomonas putida* KT2440 was used as a Nah^-^Dec^-^ plasmid-free recipient strain. The frequency of conjugative plasmid transfer was calculated as a ratio of the number of transconjugant cells to the number of donor cells.

The stability of phenotypic traits (utilization of naphthalene and decane) was determined as the percentage of transconjugant cells that retained the ability to grow on hydrocarbons compared to the total number of transconjugant cells grown on LB agar. In this case, the strains were subcultured in LB broth, with a regular (at 1-day intervals) transfer of cells to a fresh medium and the analysis of 200 transconjugants for the ability to grow on selective media with hydrocarbons.

### Assessment of growth dynamics of the strain

The abundance of microorganisms was determined using serial dilutions followed by plating on Petri dishes with LB agar. The dishes were incubated for 3 days at a temperature of 26 °C. Based on the results obtained, a microorganism growth curve was constructed. All results are derived from three independent replicates.

The specific growth rate of the microbial culture was calculated according to the biomass concentration in the phases of culture active growth according to the formula.

$$\mu \, = \,\left( {{\text{ln}}X_{{2}} {-}{\text{ln}}X_{{1}} } \right)/ \, \left( {t_{{2}} {-}t_{{1}} } \right),$$

where *X*_1_ and *X*_2_ are the biomass values corresponding to the growth times *t*_1_ and *t*_2_.

### Determination of hydrocarbon concentration

To determine the concentrations of decane and naphthalene, the hydrocarbons were extracted from the culture medium with dichloromethane (1:1, vol/vol). To determine salicylate and catechol, the aliquots of culture medium were acidified with sulfuric acid to pH 2 and filtered through a 0.22-µm filter. The samples were analyzed using the equipment of the Collective Use Center, Soil Science Faculty, and Lomonosov Moscow State University.

Decane and naphthalene were measured using a gas chromatography system (Agilent 6890, Agilent Technologies, USA) equipped with the flame ionization detector. The chromatographic column was DB-1 (30 m × 0.25 mm id, 0.25 µm). The oven temperature program was from 40 °C with an increase of 15°C /min.

Salicylate and catechol were measured using a high-performance liquid chromatography system (Agilent 1260, Agilent Technologies, USA) equipped with the UV-detector. The wavelengths: catechol, 280 nm; salicylate, 300 nm. A Synergi Hydro-RP chromatographic column (150 × 4.6 mm id, 4 µm) was used. The temperature of the column thermostat was 25 °C; the volume of the injected sample, 10 µl. Eluents: A, 90% water: 5% acetonitrile: 5% 0.1% trifluoroacetic acid; B, 95% acetonitrile: 5% 0.1% trifluoroacetic acid. Flow rate, 0.75 ml/min. Elution in gradient mode: 0 min, 5%; 15 min, 15%; 22.5 min, 40%; 25 min, 40%; 25.5 min, 95%; 30 min, 95%.

Absolute calibration with analytical standards was used for quantitation. The correlation coefficient was 0.999. The samples were diluted 100-fold before *assay*. All results are derived from five independent replicates.

*The loss of hydrocarbons* (*L*) in decane-MSEs, naphthalene-MSEs and BSEs was calculated by the following formula:

$$L = \, \left( {C_{0} {-}C_{i} } \right)/C_{0} \times {1}00 \, \left[ \% \right],$$

where *C*_0_ is initial hydrocarbon added before incubation; *C*_*i*_, residual hydrocarbon after *i* hours of growth.

*The degree of biodegradation* (*D*) *by Pseudomonas veronii* 7–41 in decane-MSEs, naphthalene-MSEs and BSEs was calculated by the following formula:

$$D = L_{{\text{m}}} {-}L_{{\text{a}}} \left[ \% \right],$$

where *L*_m_ is the loss of hydrocarbons in experimental series with microorganisms; *L*_a_, the loss of hydrocarbons in experimental series without microorganisms (abiotic control).

*The rate of consumption of the substrate* (υ) in a certain period of time was calculated by the following formula:

$$\upsilon = \left[ {\left( {C_{{{\text{mT2}}}} - C_{{{\text{mT1}}}} } \right) - \left( {C_{{{\text{aT2}}}} - C_{{{\text{aT1}}}} } \right)} \right]/\left( {T_{2} - T_{1} } \right)\,\,\left[ {{\text{mg}}/\left( {{\text{l}} \times {\text{h}}} \right)} \right]$$

where *T*_2_, *T*_1_ is time (hours); *C*_m T2_, *C*_m T1_ is the residual concentration of hydrocarbons at time *T*_2_ or *T*_1_ in the experimental series with microorganisms;

*C*_a T2_, *C*_a T1_ is the residual concentration of hydrocarbons at time *T*_2_ or *T*_1_ in the experimental series without microorganisms (abiotic control).

### Enzyme activity

Enzyme activity was measured in a cell-free extract obtained from cell biomass. Therefore, this parameter could be studied only starting from the middle of the exponential growth phase. The biomass was washed twice with chilled 0.05 M phosphate buffer (pH 7.0) and resuspended in 0.02 M phosphate buffer (pH 7.5). Cell suspension (5 ml sample) was subjected to sonication (using an MSE150 disintegrator) for 1.5 min (3 × 30 s) at 4 °C. Cell debris was removed by centrifugation (Rotanta 460R centrifuge, Hettich Zentrifugen, Germany) for 20 min at 10,000*g* and 4 °C. The supernatant was used as a cell-free extract. Activities of the enzymes were determined as previously described for naphthalene 1.2-dioxygenase in Dua and Meera^60^, for salicylate hydroxylase in Yamamoto^61^, for alkane hydroxylase in Shweta Mishra^62^, for catechol 1.2-dioxygenase in Hegeman^63^, and for catechol 2.3-dioxygenase in Feist and Hegeman^64^. The protein concentration was determined according to Bradford^65^.

## Supplementary Information

Supplementary Information.

## Data Availability

The datasets presented in this study can be found in online repositories. The names of the repository/repositories and accession number(s) can be found here: https://www.ncbi.nlm.nih.gov/genbank/, CP089551; https://www.ncbi.nlm.nih.gov/genbank/, CP089552.
