# Supplementary material for: Pseudomonas veronii strain 7–41 degrading medium-chain n-alkanes and polycyclic aromatic hydrocarbons
Source: Sci Rep. 2022 Nov 28;12:20527. doi: 10.1038/s41598-022-25191-5 (PMC9705281; doi:10.1038/s41598-022-25191-5)
Supplement: Supplementary file 1 — Supplementary Information. [file 41598_2022_25191_MOESM1_ESM.pdf]

Supplementary material

***Pseudomonas veronii* strain 7-41 degrading medium-chain *n*-alkanes and polycyclic aromatic hydrocarbons**

Mullaeva S.A.[1], Delegan Ya. A.[1,2], Streletskii R.A.[3], Sazonova O.I.[1], Petrikov K.V.[1], Ivanova A.A.[1], Dyatlov I. A.[2], Shemyakin I.G.[2], Bogun A.G.[2], Vetrova A.A.[1,*]

[1] Federal Research Center «Pushchino Scientific Center for Biological Research of the Russian Academy of Sciences», G.K. Skryabin Institute of Biochemistry and Physiology of Microorganisms of the Russian Academy of Sciences, Pushchino, Moscow Region, 142290, Russian Federation
[2] State Research Center for Applied Microbiology and Biotechnology, Obolensk, 142279, Russian Federation
[3] Faculty of Soil Science, Laboratory of Ecological Soil Science, Lomonosov Moscow State University, Moscow, 119991, Russian Federation

*- corresponding author Vetrova A.A. – phdvetrova@gmail.com.

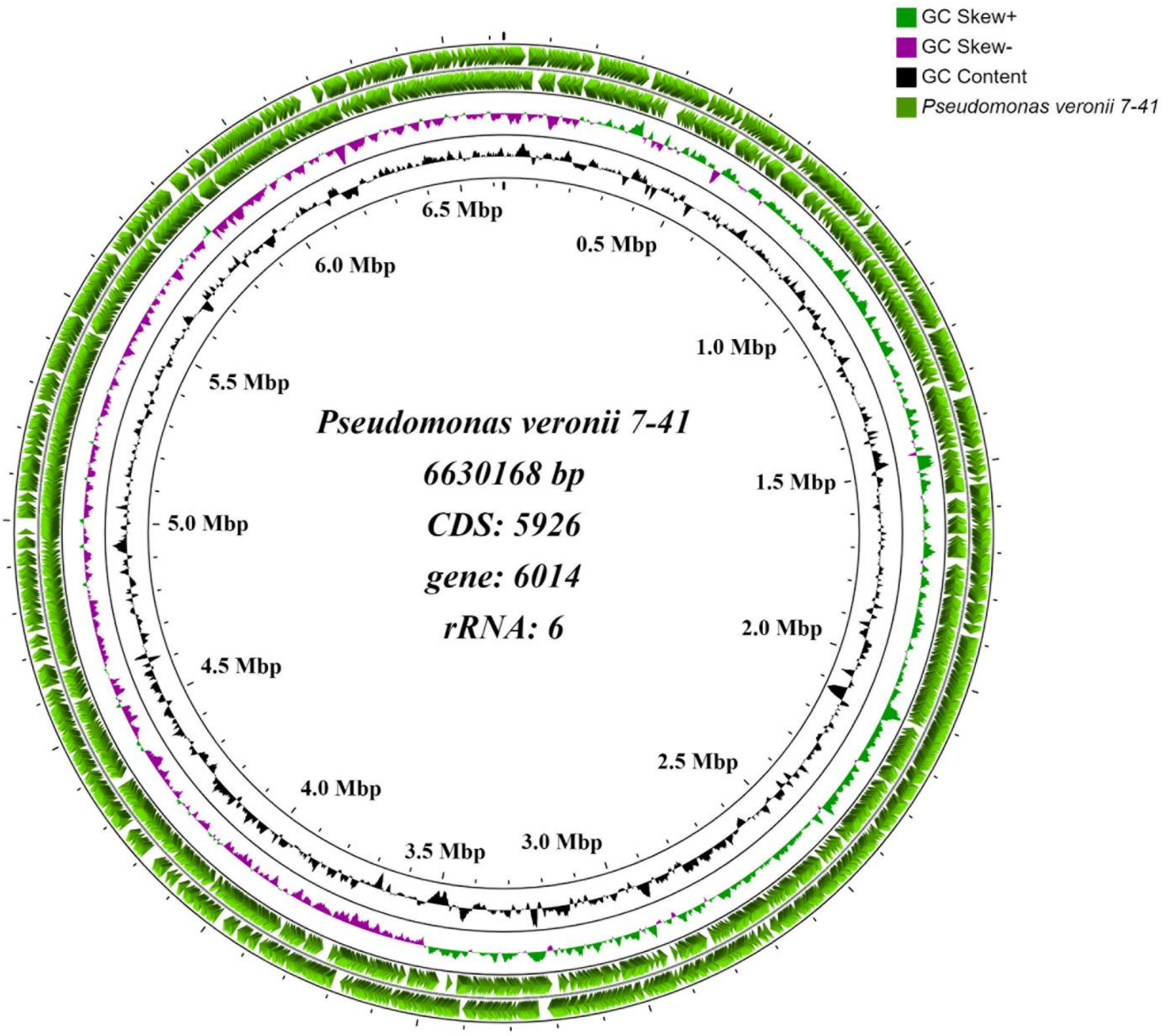

Fig. 1S. Circular map of the *Pseudomonas veronii* 7-41 chromosome. From outside to the center: all CDS and RNA genes on forward strand, all CDS and RNA genes on reverse strand, GC content, and GC skew.

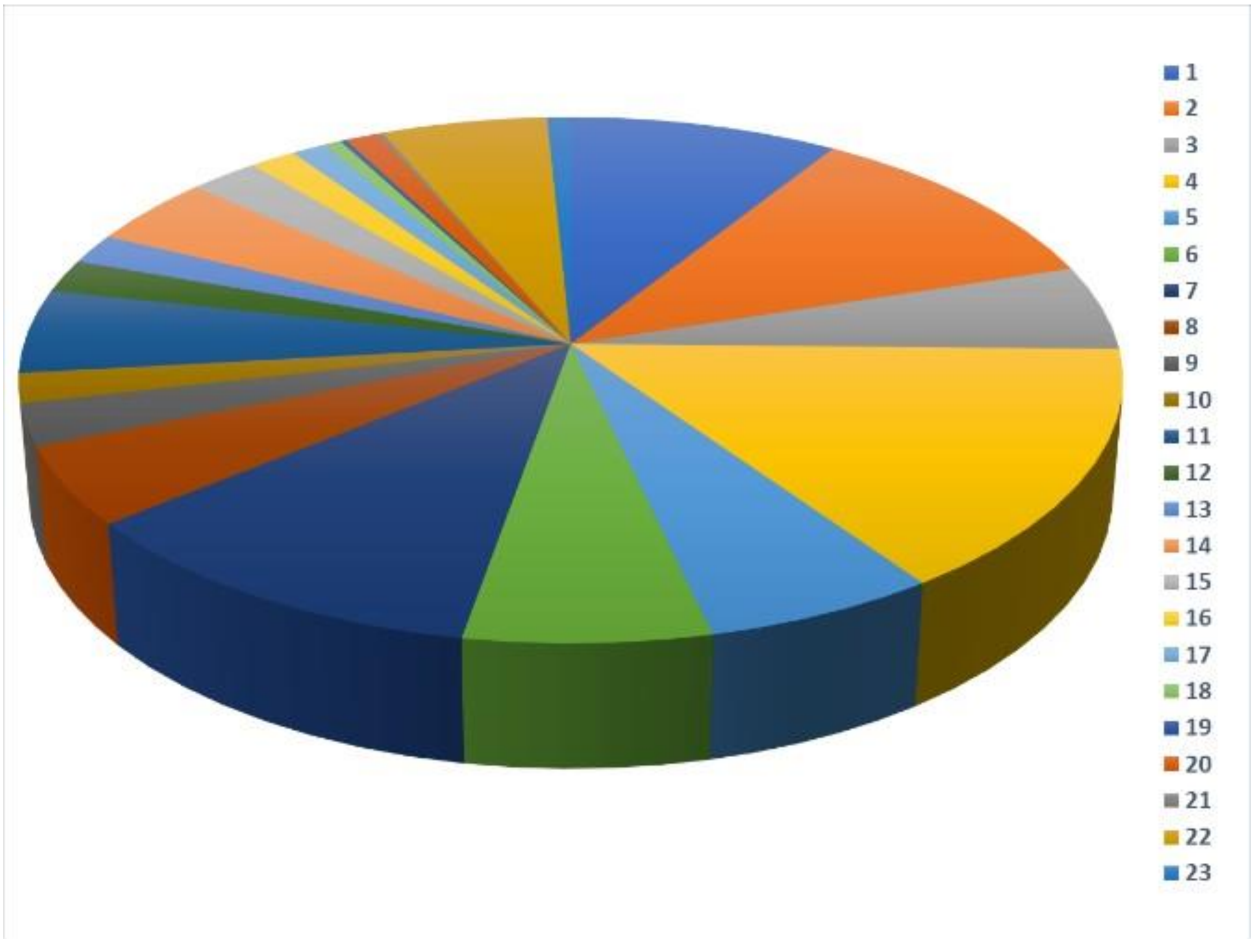

Fig. 2S. Genes associated with general COG functional categories (KEGG function classification): 1. Carbohydrate metabolism; 2. Protein families: signaling and cellular processes; 3. Genetic information processing; 4.Environmental information processing; 5.Unclassified: metabolism; 6.Amino acid metabolism; 7.Protein families: genetic information processing; 8.Metabolism of cofactors and vitamins; 9.Nucleotide metabolism; 10.Unclassified: genetic information processing; 11.Cellular processes; 12. Lipid metabolism; 13. Protein families: metabolism; 14. Energy metabolism; 15. Unclassified: signaling and cellular processes; 16. Glycan biosynthesis and metabolism; 17. Metabolism of other amino acids; 18.Metabolism of terpenoids and polyketides; 19.Organismal systems; 20. Biodegradation of xenobiotics; 21. Biosynthesis of other secondary metabolites; 22. Human diseases; 23. Unclassified.

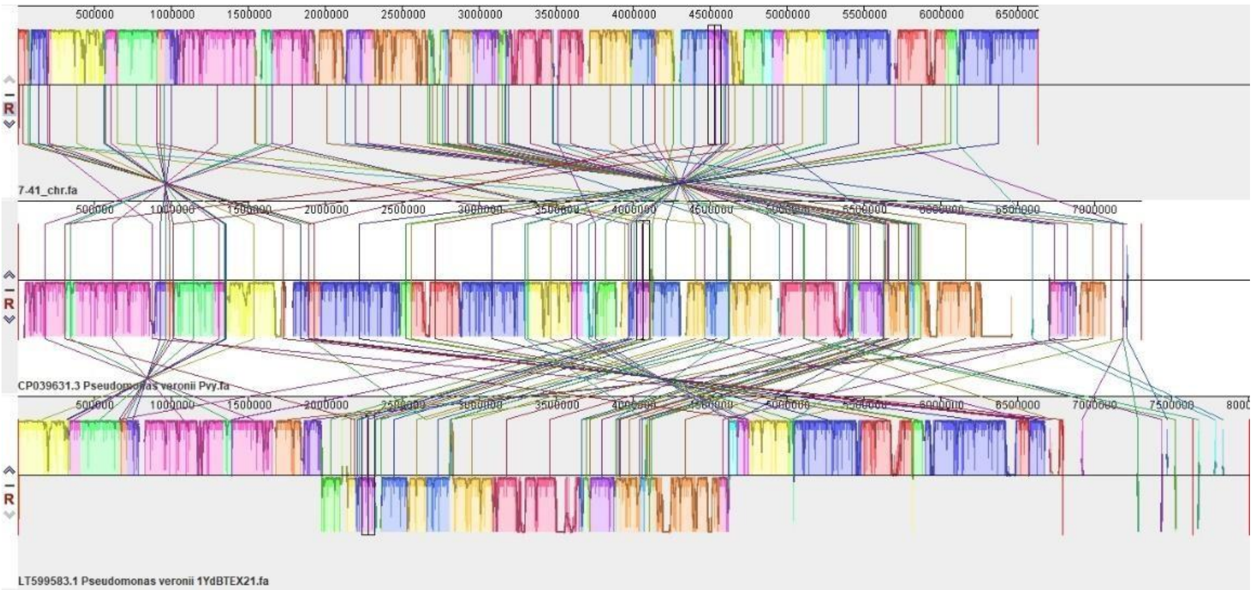

Fig.3S. Mauve visualization of locally collinear blocks identified between chromosomes of *Pseudomonas veronii* 7-41, *Pseudomonas veronii* Pvy and *Pseudomonas veronii* 1YdBTEX21. Vertical bars demarcate interchromosomal boundaries (the homology regions of the three chromosomes)

Table 1S. Similarity of predicted gene products from the *nah*- and *alk*- gene clusters of plasmid pCP7-41 (P. veronii 7-41) to selected homologs.

p7-41	Position (bp)[a]	GC%	Proposed function	No. of amino acids, pCP7-41/ relative[b]	% Amino Acid identity	Source microorganism	GeneBank accession number[c]
naphthalene catabolic genes							
nahD	36911..37513c	46.6	2-hydroxychromene-2-carboxylate isomerase	200/203	78	*Pseudomonas putida* plasmid NAH7	WP_011475384.1
				200/199	78	*Pseudomonas veronii* strain Pvy plasmid unnamed	WP_011117407.1
				200/200	100	*Pseudomonas veronii* VI4T1	OPK04001.1
nahE	37718..38722c	52	1, 2-dihydroxybenzylpyruvate aldolase	334/334	92	*Pseudomonas stutzeri*	AAD02141.1
				334/331	89	*Pseudomonas putida* plasmid NAH7	WP_011475383.1
				334/334	100	*Pseudomonas veronii* VI4T1	OPK04000.1
nahQ	38768..39403c	48.3	Outer membrane beta-barrel protein	211/180	83	*Pseudomonas frederiksbergensis*	WP_235864979.1
				211/211	80	*Pseudomonas veronii* strain Pvy plasmid unnamed	WP_012727770.1
				211/215	80	*Pseudomonas putida* plasmid pDTG1	WP_228409390.1
				211/210	77	*Pseudomonas putida* plasmid NAH7	WP_011475382.1
				211/211	99	*Pseudomonas veronii* VI4T1	OPK03999.1
nahC	39476..40384c	50.5	1,2-dihydroxynaphthalene dioxygenase	302/302	92	*Pseudomonas putida* plasmid pDTG1	WP_011117404.1
				302/302	92	*Pseudomonas veronii* strain Pvy plasmid unnamed	WP_141123765.1
				302/302	100	*Pseudomonas veronii* VI4T1	OPK03998.1
nahF	40412..41863c	53.6	Salicylaldehyde dehydrogenase	483/483	100	*Pseudomonas veronii* VI4T1	OPK03997.1

				483/483	91	*Pseudomonas putida* plasmid NAH7	WP_011475380.1
				483/483	92	*Pseudomonas veronii* strain Pvy plasmid unnamed	WP_155678977.1
				483/483	93	*Pseudomonas* sp. MPDS	WP_173407259.1
nahB	41910..42689c	49.4	Naphthalene cis-dihydrodiol dehydrogenase	259/259	100	*Pseudomonas veronii* VI4T1	OPK03996.1
				259/259	93	*Pseudomonas veronii* strain Pvy plasmid unnamed	WP_141123764.1
				259/259	91	*Pseudomonas putida* plasmid NAH7	WP_011475379.1
				259/275	91	*Pseudomonas putida*	AFM52767.1
nahAd	42759..43340c	50.2	Naphthalene 1,2-dioxygenase small subunit	193/193	100	*Pseudomonas veronii* VI4T1	OPK03995.1
				193/193	85	*Pseudomonas stutzeri*	WP_020307249.1
				193/193	83	*Pseudomonas putida* plasmid NAH7	WP_011475378.1
				193/193	83	*Pseudomonas veronii* strain Pvy plasmid unnamed	WP_141123763.1
nahAc	43356..44705c	49.5	Naphthalene 1,2-dioxygenase large subunit	449/449	100	*Pseudomonas veronii* VI4T1	OPK03994.1
				449/449	94	*Pseudomonas putida* plasmid NAH7	WP_011475377.1
				449/449	93	*Pseudomonas veronii* strain Pvy plasmid unnamed	WP_155678976.1
nahAb	44758..45084c	47.7	Naphthalene 1,2-dioxygenase ferredoxin	108/108	100	*Pseudomonas veronii* VI4T1	OPK03993.1
				108/107	89	*Pseudomonas putida* plasmid NAH7	WP_011475376.1
				108/104	89	*Pseudomonas veronii* strain Pvy plasmid unnamed	WP_009399901.1

nahAa	45212..46198c	54.2	Naphthalene 1,2-dioxygenase reductase	328/328	100	*Pseudomonas veronii* VI4T1	OPK03992.1
				328/328	84	*Pseudomonas putida* plasmid NAH7	WP_011475375.1
				328/328	89	*Pseudomonas veronii* strain Pvy plasmid unnamed	WP_155678975.1
nahR	51275..52177c	56.6	LysR-type transcriptional regulator	300/300	100	*Pseudomonas veronii* VI4T1	OPK05586.1
				300/374	82	*Pseudomonas putida* plasmid NAH7	WP_011475385.1
				300/300	95	*Pseudomonas veronii* strain Pvy plasmid unnamed	WP_003292087.1
nahG	52326..53630	58.5	Salicylate 1-monooxygenase	434/434	100	*Pseudomonas veronii* VI4T1	OPK05587.1
				434/434	86	*Pseudomonas putida* plasmid NAH7	WP_011475386.1
				434/437	86	*Pseudomonas veronii* strain Pvy plasmid unnamed	WP_043221467.1
nahT	54066..54386	53.6	Chloroplast ferredoxin-like protein	106/106	100	*Pseudomonas veronii* VI4T1	OPK05588.1
				106/108	78	Pseudomonas *putida* plasmid NAH7	WP_011475387.1
				106/112	88	*Pseudomonas veronii* strain Pvy plasmid unnamed	WP_003292091.1
nahH	54401..55324	52.5	Catechol 2,3-dioxygenase	307/307	100	*Pseudomonas veronii* VI4T1	OPK05589.1
				307/307	87	*Pseudomonas putida* plasmid NAH7	WP_011475388.1
				307/307	89	*Pseudomonas veronii* strain Pvy plasmid unnamed	WP_009397178.1
nahI	55359..56819	63.7	2-Hydroxymuconic semialdehyde dehydrogenase	486/486	100	*Pseudomonas veronii* VI4T1	OPK05590.1
				486/486	94	*Pseudomonas putida* plasmid NAH7	WP_011475389.1
				486/486	91		WP_009397179.1

						Pseudomonas veronii strain Pvy plasmid unnamed	
nahN	56827..57696	65.3	2-hydroxymuconic semialdehyde hydrolase	289/293	99	*Pseudomonas veronii* VI4T1	OPK05599.1
				289/293	79	*Pseudomonas putida* plasmid NAH7	WP_011475390.1
				289/287	83	*Pseudomonas veronii* strain Pvy plasmid unnamed	WP_009397180.1
nahL	57706..58491	64.6	2-oxypent-4-enoate hydratase	261/261	99	*Pseudomonas veronii* VI4T1	OPK05591.1
				261/261	97	*Pseudomonas putida* plasmid NAH7	WP_011475391.1
				261/261	87	Pseudomonas veronii strain Pvy plasmid unnamed	WP_009397181.1
nahO	58512..59435	64.7	Acetaldehyde dehydrogenase	307/307	100	*Pseudomonas veronii* VI4T1	OPK05592.1
				307/307	97	*Pseudomonas putida* plasmid NAH7	WP_011475392.1
				307/307	94	*Pseudomonas veronii* strain Pvy plasmid unnamed	WP_009397182.1
nahM	59449..60489	63.1	4-hydroxy-2-oxovalerate aldolase	346/346	100	*Pseudomonas veronii* VI4T1	OPK05593.1
				346/346	96	*Pseudomonas putida* plasmid NAH7	WP_011475393.1
				346/346	94	*Pseudomonas veronii* strain Pvy plasmid unnamed	WP_003450283.1
nahK	60486..61280	62.6	4-oxalocrotonate decarboxylase	264/264	100	*Pseudomonas veronii* VI4T1	OPK05594.1
				264/264	97	*Pseudomonas putida* plasmid NAH7	WP_011475394.1
				264/264	91	*Pseudomonas veronii* strain Pvy plasmid unnamed	WP_003292101.1
nahJ	61335..61526	57.8	4-oxalocrotonate tautomerase	63/63	100	*Pseudomonas veronii* VI4T1	OPK05595.1
				63/63	85	*Pseudomonas putida* plasmid NAH7	WP_011475395.1

				63/63	79	*Pseudomonas veronii* strain Pvy plasmid unnamed	WP_003292102.1
nahX	61564..62004	62.6	heme-binding protein	146/146	100	*Pseudomonas veronii* VI4T1	OPK05596.1
				146/140	70	*Pseudomonas putida* plasmid NAH7	WP_011475396.1
				146/146	83	*Pseudomonas veronii* strain Pvy plasmid unnamed	WP_003292104.1
				n-alkane catabolic genes			
alkL	67882..68574c	41	Outer membrane beta-barrel protein	230/230	100	*Pseudomonas veronii* VI4T1	OPK05683.1
				230/230	100	*Pseudomonas putida* P1	CAB69081.1
				230/230	80	*Pseudomonas putida* strain GPo1 (OCT plasmid)	CAB54056.1
alkK	68680..70320c	45.5	long-chain-fatty-acid-CoA ligase	546/546	100	*Pseudomonas veronii* VI4T1	OPK05682.1
				546/546	99	*Pseudomonas putida* P1	CAB69080.1
				546/546	80	*Pseudomonas putida* strain GPo1 (OCT plasmid)	CAB54055.1
alkJ	70405..72063c	43.7	Alcohol dehydrogenase	552/552	100	*Pseudomonas veronii* VI4T1	OPK05681.1
				552/552	100	*Pseudomonas putida* P1	CAB51051.1
				552/558	85	*Pseudomonas putida* strain GPo1 (OCT plasmid)	CAB54054.1
alkH	72102..73553c	44.1	Aldehyde dehydrogenase	483/483	100	*Pseudomonas veronii* VI4T1	OPK05680.1
				483/483	100	*Pseudomonas putida* P1	CAB51050.1
				483/483	80	*Pseudomonas putida* strain GPo1 (OCT plasmid)	CAB54053.1
alkG	73595..73786c	47.9	Rubredoxin-2	175/175	98	*Pseudomonas veronii* VI4T1	OPK05679.1
				175/175	98		CAB51049.1

				175/173	47	Pseudomonas putida P1<br><br>*Pseudomonas putida* strain GPo1 (OCT plasmid)	CAB54052.1
alkF	74163..74567c	43.5	Rubredoxin-1	134/134	100	*Pseudomonas veronii* VI4T1	OPK05678.1
				134/134	100	*Pseudomonas putida* P1	CAB51048.1
				134/132	47	*Pseudomonas putida* strain GPo1 (OCT plasmid)	CAB54051.1
alkB	74850..76058c	48.2	Alkane 1-monooxygenase	402/402	100	*Pseudomonas veronii* VI4T1	OPK05677.1
				402/402	100	*Pseudomonas putida* P1	CAB51047.1
				402/401	92	*Pseudomonas putida* strain GPo1 (OCT plasmid)	CAB54050.1
alkS	79028..81502	45.5	alk gene regulator	824/883	99	*Pseudomonas veronii* VI4T1	OPK05675.1
				824/883	83	*Pseudomonas putida* P1	CAB69079.1
				824/882	99	*Pseudomonas putida* strain GPo1 (OCT plasmid)	CAB54064.1
alkT	81551..82708	47.8	Rubredoxin-NAD(+) reductase	385/385	100	*Pseudomonas veronii* VI4T1	OPK05674.1
				385/385	100	*Pseudomonas putida* P1	CAB69078.1
				385/385	92	*Pseudomonas putida* strain GPo1 (OCT plasmid)	CAB54063.1

[a] Letters indicate coding strand: c, complementary strand; d, direct strand.
[b] Number of amino acids in p7-41 CDS/number in the closest relative.
[c] GenBank accession number of the closest relative protein.

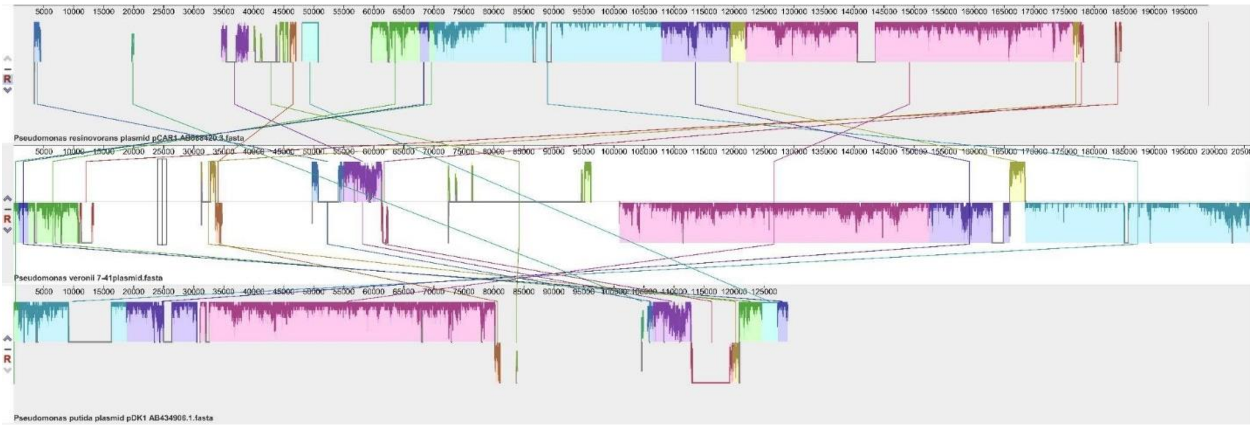

Fig.4S. Mauve visualization of locally collinear blocks identified between plasmids of *P. veronii* 7-41, *P. putida* HS1 and *P. resinovorans* strain CA10. Vertical bars indicate interplasmid boundaries.
